# Fetal Pancreatic Hamartoma Associated with Hepatoblastoma—An Unusual Tumor Association

**DOI:** 10.3390/diagnostics12030758

**Published:** 2022-03-20

**Authors:** Valentin Varlas, Oana Neagu, Andreea Moga, Radu Bălănescu, Roxana Bohiltea, Radu Vladareanu, Laura Balanescu

**Affiliations:** 1 Department of Obstetrics and Gynecology, Filantropia Clinical Hospital, 011171 Bucharest, Romania; valentin.varlas@umfcd.ro; 2 Faculty of General Medicine, “Carol Davila” University of Medicine and Pharmacy, 37 Dionisie Lupu St., 020021 Bucharest, Romania; radu.balanescu@umfcd.ro (R.B.); vladareanu@gmail.com (R.V.); laura.balanescu@umfcd.ro (L.B.); 3 Department of Anatomopathology, “Carol Davila” University of Medicine and Pharmacy, 050474 Bucharest, Romania; oana.neagu@yahoo.com; 4 Department of Pediatric Surgery, Children Emergency Hospital “Grigore Alexandrescu”, 011743 Bucharest, Romania; alecsandra.moga@drd.umfcd.ro; 5 Department of Obstetrics and Gynecology, Elias Clinical Hospital, 011461 Bucharest, Romania

**Keywords:** fetal abdominal mass, fetal pancreatic tumors, hamartoma, hepatoblastoma, ultrasonography, fetal inferior vena cava anomalies

## Abstract

Abdominal tumor masses are a very rare disease in the fetus. The authors present the first reported case of neonatal multicystic adenomatoid hamartoma of the pancreas associated with well-differentiated fetal epithelial subtype hepatoblastoma and reveal clinical, histologic, and imagistic aspects. Case presentation: A 36-week-old female newborn in whom a 25-week ultrasound showed a relatively homogeneous pancreatic echogenic mass (34 × 30 × 55 mm) with compression of the inferior vena cava and retrograde dilation. Postnatal CT showed a giant pancreatic tumor mass (113 × 70 × 60 mm), with areas enhancing contrast and cystic/necrotic areas and a hypodense, hypocaptive nodule of 8 × 6 mm located at segment IV of the liver; thrombosis of the subhepatic segment of the inferior vena cava and both renal veins. Histopathological and immunohistochemical studies confirmed the diagnosis of multicystic pancreatic adenomatoid hamartoma and well-differentiated fetal epithelial subtype hepatoblastoma. Conclusions: Pancreatic hamartoma can be difficult to diagnose (especially prenatal), with or without nonspecific symptoms. The synchronous presence of hepatoblastoma complicated the therapeutic conduct and prognosis of this case, with the diagnosis being confirmed histopathologically and immunohistochemically after liver biopsy.

## 1. Introduction

Fetal intra-abdominal complex tumors are extremely rare and are benign in most cases. Likewise, tumors of the fetal pancreas are extremely rare and mainly represent congenital islet cell adenoma [1]. The authors report a unique case of pancreatic hamartoma associated with a hepatoblastoma in a 36-week-old newborn who presented an abdominal mass. A literature review did not identify any previous reports of the association of these conditions in a newborn. The case is all the more interesting as suspicion of the pancreatic tumor in the fetus was raised by imagistic findings from the 25th week of pregnancy. The prevalence of congenital tumors is 7.2 per 100,000 live births [2]. The incidence of fetal neoplasms that can be detected by ultrasound is 36.5 per million live births, according to the study by Solt et al. [3]. Hepatoblastoma is the most common liver malignancy, with an increased incidence in the first year of life, representing 11.2 cases per 1 million [4]. The incidence in Europe is 1.2 per million [5] and in the USA between 0.8–1.5 per million [6], with over 90% occurring before the age of 5, and can be detected by ultrasound in the fetus. In contrast, only nine cases of pancreatic hamartoma have been reported in newborns and children in the literature [7,8].

The purpose of this case report was to show the rare occurrence of synchronous pancreatic hamartoma associated with hepatic hepatoblastoma and to report the associated imaging and infant outcome.

## 2. Case Report

**Figure 1:**
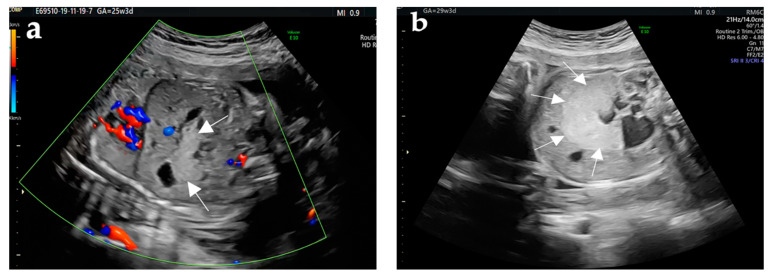

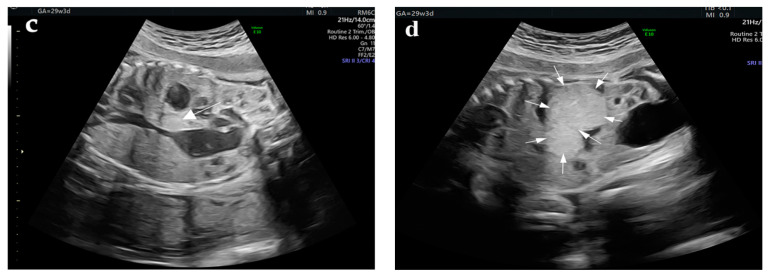
Evolution of ultrasonography findings of a pancreatic tumor in a 25-week-old fetus (**a**) and at 29 weeks (**b**,**c**). (**a**) Sagittal image shows an echogenic mass (arrows) with a well-differentiated contour in the fetal pancreas. (**b**) Transversal image revealed an increased echogenic mass at 29 weeks in the fetal pancreas (arrows) compared to the previous examination. (**c**) Coronal image shows compression of the inferior vena cava by the tumor, with retrograde dilation. (**d**) Longitudinal image revealed the pancreatic tumor.

We present the case of a premature female newborn at 36 weeks from a 32-year-old mother with two previous births. The pregnancy had a normal course until the 25th week, when an ultrasound scan revealed a pancreatic echogenic mass (34 × 30 × 55 mm), relatively homogeneous, with compressed inferior vena cava (IVC), normal amniotic fluid, lungs, liver, and kidneys (Table 1 and Figure 1a–d). Preterm birth at 36 weeks was triggered spontaneously by PPROM, and the baby was born by cesarean section, with a birth weight of 2800 g suitable for gestational age and Apgar score of 8 and 9, respectively, at 1 and 5 min. At birth, the physical examination of the newborn revealed the presence of a firm mass in both the right and left quadrants of the abdomen. Shortly after birth, the newborn developed a mild respiratory distress syndrome that required oxygen therapy. The infant also had hypoglycemia and hypocalcemia, requiring intravenous glucose and calcium. Lab exam revealed a higher CEA and normal AFP levels.

The patient had been admitted to the pediatric surgery department, Emergency Children Hospital “Grigore Alexandrescu” Bucharest, Romania, where the following exams were performed (Table 2).

**Table 2:** Synopsis of postnatal imagistic findings.

	Type of Exam	Age	Imagistic Findings
Preoperative	Lung radiograph	2nd day	ascension of the diaphragm due to a pancreatic tumor mass (Figure 2a)
	CT of the thorax, abdomen, and pelvis	6th day	giant pancreatic tumor mass (113 × 70 × 60 mm), with areas enhancing contrast and cystic/necrotic areas that encase mesenteric artery and vein, splenic vein, and porta inhomogeneous liver—hypodense, hypocaptive nodule of 8 × 6 mm located in segment IV spleen and kidneys—normal aspect; no pulmonary or bone lesions thrombosis of the subhepatic segment of the IVC and renal veins (Figure 2b)
Postoperative	CT of abdomen and pelvis	Three weeks	reduction of the retroperitoneal tumor masses (40 × 34 × 28 mm) compared to the previous examination, keeping the morphological characters mentioned extensive thrombosis of the IVC, with dilatation of the vein up to 30 mm, and of the renal veins significant aortic parietal edema, with lumen stenosis up to 2.2 mm (Figure 2c) ascites in large quantity
	CT of the brain, thorax, abdomen, and pelvis	Four weeks	*brain*—no metastatic lesion *thorax*—condensation processing in the posterior segments bilateral, small bubbles of emphysema in the apical segment of the left inferior segment and the posterior-basal segment of the right inferior lobe, no pleural or pericardial effusion *abdomen and pelvis* ▪ inhomogeneous liver with two native hypodense masses without enhancement of contrast (segment IV a—11 × 6 mm, segment V—12 × 7 mm). Inhomogeneous tumor mass located in the root of the mesentery, diffuse, hypodense, with iodophilic tissue areas of 42 × 41 × 29 mm. The infra hepatic segment of the IVC increased to 35 mm ▪ extensive thrombosis in the splenic, right renal, mesenteric vein, port-mesenteric junction, with a thrombus of 16 × 10 mm, posterior to the aorta (stenosis at this level) ▪ aorta with decreased size in the infrarenal segment (2–3 mm) ▪ ascites in high quantity (52 mm anteroposterior in the inferior abdomen) ▪ inhomogeneous spleen with diffuse hypodense areas (perfusion disturbances) *bone*—no lesions in the examined areas
	Abdominal ultrasound	Five weeks	the retrohepatic segment of the IVC has 22 mm, with an 18 mm thrombus umbilical area—inhomogeneous abdominal tumor mass, 51 mm anteroposterior diameter, with multiple calcifications small to moderate ascites around the liver, spleen, and in the lower abdominal floor
	Abdominal ultrasound	Seven weeks	the IVC diameter 31 mm with a 37 × 17 mm thrombus hypoechoic nodular formation 46 × 34 mm in the epigastrium, relatively well-delimited, inhomogeneous with transonic areas inside, moderately vascularized centrally without ascites or other modifications
	Abdominal ultrasound	Three months + three weeks	liver—right lobe 81mm, relatively homogeneous, with a nodular image of 23 mm with mixed appearance, predominantly transonic in segment VII IVC diameter 40 mm; it has a 37 mm intraluminal transonic image, with numerous echoes (Figure 2e,f)
	Full-body CT	Six months	multiple hepatic tumors with cystic components, and no pancreatic tumor (Figure 3) extreme fusiform dilatation of the IVC (13.3 × 8.3 × 7.7 cm), the left renal vein (11.7 × 9.3 × 7.4 cm), and the superior mesenteric vein, with no distal flow with the development of distal collateral circulation. All dilated vascular structures presented thrombotic areas (Figure 2d)
	Last CT	Nine months	increased size of the tumor liver and IVC diameter; significant dilation of the left renal vein

**Figure 3:**
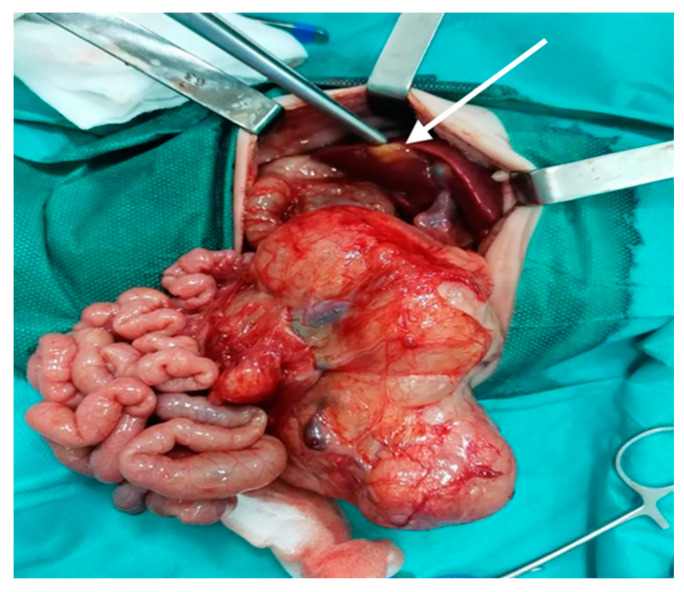
Intraoperative image of pancreatic hamartoma and hepatic tumor (arrow).

The laparotomy was performed at 1 week old. Intraoperative findings were as follows: giant tumor mass on the anterior face/inferior border of the pancreas, 15 × 10 cm, polylobate, firm, with cystic areas, strongly adherent to the mesentery, duodenum, colon, ileal loops on which has a mass effect; nodular mass in the Vth segment of the liver, under the hepatic capsule, size < 1 cm, firm consistency suggestive for metastatic nodule; mesentery tumor mass, 1 × 1 cm, with a consistency similar to the pancreatic tumor (Figure 3).

The surgery consisted of partial resection of the pancreatic tumor and complete resection of the mesenteric tumor. Dissection of the tumor was very difficult due to the intratumoral trajectory of the superior and inferior mesenteric vessels and the intimate contact with the inferior vena cava but without invading it; also, the tumor penetrated the retroperitoneal space without invading the kidneys and ureters. Subtotal resection was carried out with effraction of the tumor capsule, and a small area of the pancreas was preserved. The recovery after surgery was complicated; the patient was intubated, vasopressor treatment and insulin therapy were required. On the 12th day after the operation, the increase in the size of the abdomen required intraperitoneal drainage with the evacuation of chylous ascites.

The initial surgical histopathological examination revealed a low-malignancy serous cystadenocarcinoma (Table 3).

The subsequent monitoring of the patient was performed in the Pediatric Oncology Department of the Oncological Institute «Prof. Dr. Al. Trestioreanu». Clinical examination at admission: afebrile, acceptable general condition, pallor, abdominal distension, peritoneal drainage approx. 20–30 mL/24 h, breast and bottle feeding, weight = 2700 g; laboratory findings: LDH = 2×, GGT = 4×. At the age of 2 months, the patient received a round of chemotherapy (FOLFIRINOX protocol), because the initial pathological examination was serous cystadenocarcinoma, and he received Leucovorin (400 mg/m^2^) with a total dose of 30 mg and 5-FU 400 mg/m^2^, then 1200 mg/m^2^ (a total dose of 118 mg). After reevaluation of the histopathological stains, the final diagnosis showed that the resected tumor was, in fact, a pancreatic mixed hamartoma (solid and cystic), a benign tumor that does not require chemotherapy.

The patient needed repeated blood transfusions in the following months due to anemia. At the last CT examination performed at 9 months old, there was an increase in liver tumor mass, and a liver biopsy was performed. The histopathological diagnosis was well-differentiated fetal subtype hepatoblastoma (Table 3).

**Table 3:** Synopsis of histological evaluation.

Type of Exam	Histopathological Findings
The histopathological exam with immunohistochemical tests (*surgical specimen*)	pancreatic tumor: low-malignancy serous cystadenocarcinoma with ductal and acinar appearance areas, without excluding atypical serous cystadenoma. AFP−, Ki67+ in 15% of epithelial and stromal cells. CD30—(excludes embryonic carcinoma).
Histopathological re-evaluation (Dr. Monique Fabre, Necker Hospital, Paris, France)	mixed pancreatic tumor: ▪ solid component—dilated nests of acini and ducts with lobular organization and, more frequently, disorganized (dilated acini that incorporate ducts of different sizes). ▪ multicystic component—cysts with different dimensions and imprecisely delimited, bordered by unilayered mucinous cells with biliary–pancreatic differentiation, with a slight tendency to pseudo-stratification. Focal nests form multipapillary structures. areas of nuclear basophilia, no mitosis or infiltrative growth. The confluence of these cysts could result in a unilocular cyst bordered by thin epithelium in most areas, with no pancreatic tissue between the cysts. Linear eosinophilic calcifications are present. Inflammatory infiltrates present intracystic. There are necrosis areas, acini appear to be connected to cysts, dilated, sometimes with a tubular appearance. The cells show marked eosinophilic granules in the apical area, basophilic staining at the base, and large nuclei (Figure 4a,b). epithelial component expresses CK7+++ (apical, basal and lateral), CK8/18+ (diffuse, in the cyst epithelium, CK19+++ (cytoplasm with apical accentuation), CK20+ (apical, focal), CDX2+ (focal). no clear small cell component suggests serous differentiation, no rich subepithelial capillary network inferior to cysts—a feature in serous cystadenoma. HE staining cannot identify the endocrine component, Ki67+, in 15% (including epithelial and stromal cells) (Figure 5a–c). stroma has an inhomogeneous structure, and there is paucicellular connective tissue between the cysts, hyalinized, in various quantities. Abundant inflammatory infiltrates present throughout the tumor mass and edematous stroma without fatty components or calcifications. Some unilocular lesions have hyaline walls of various sizes. on some samples, a thin pseudo-fibrous capsule delimits the tumor tissue from a thin area of atrophic pancreatic tissue or peritoneum. There is no hyperplasia of the pancreatic islet cells without chronic pancreatic obstruction in the remaining pancreatic tissue. At the edge of the tumor, remaining atrophic pancreatic tissue, a rich network of large vessels (arteries and veins) and nerves with no tumor infiltration. Proposed diagnosis: mixed hamartoma (solid and cystic), also called multicystic adenomatoid hamartoma of the pancreas; benign lesion.
The histopathological exam with immunohistochemical tests *(liver tumor biopsy)*	biopsy fragments of fibroconjunctive tissue with tumor proliferation composed of round cells, pale eosinophilic or clear cytoplasm, central nucleus, small in size, normochromic; tumor cells are arranged in nests, cords, trabeculae; some cells contain brown pigment; very low mitotic rate. glypican+; OCH1E5+; INI-1+ in tumor cell nuclei; αFP−; β Catenin + membrane and cytoplasmic; Ki67+ 10% in tumor cells. Proposed diagnosis: well-differentiated fetal epithelial subtype hepatoblastoma.
Autopsy	nests of hepatocytes of the developing fetal liver, separated by fibrous or myxoid edematous stroma; thin trabeculae of hepatocytes forming pseudo acinar spaces with central bile plug (Figure 4c,d). vena cava wall: granulation tissue with neoangiogenesis and giant thrombus (Figure 4e,f)

**Figure 6:**
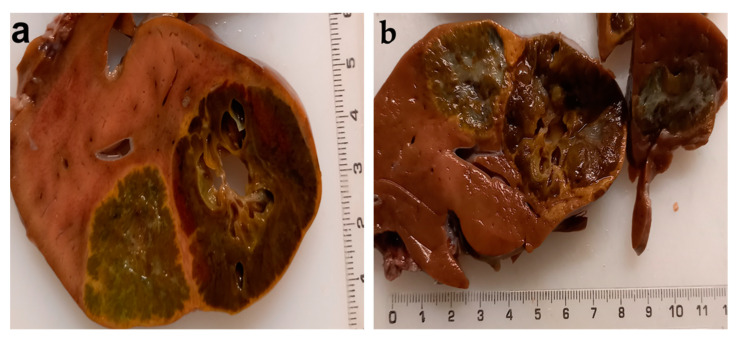
(**a**,**b**) Postmortem macroscopic pathology shows liver damage (hepatoblastoma).

At 11 months old, the patient died at home at 2 months after discharge from the hospital. An autopsy revealed the presence of multiple hepatic tumors with cystic components (hepatoblastoma) (Figure 6a,b).

## 3. Discussion

The complex tumor masses encountered in the newborn continue to be a real challenge from a clinical, diagnostic, therapeutic, and prognostic point of view. This is due to the low number of cases reported in the literature. The peculiarity of this case was determined both by the synchronous occurrence of multicystic adenomatoid pancreatic hamartoma and hepatoblastoma in a 1-week-old newborn.

Pancreatic hamartomas are extremely rare tumors in children and even more so in newborns. A systematic search of the literature identified a total of forty-five cases of pancreatic hamartomas, of which thirty-six were in adults, six in children, and three in newborns [9,10]. Two of the six children and one of the three newborns had trisomy 18 [7,8,9,10,11] (Table 4).

In prenatal life, abdominal tumors of the fetus are rare and sometimes difficult to diagnose. Although currently, there is no detailed and unanimously accepted classification of fetal tumors, the antenatal diagnosis of intra-abdominal tumors regarding their origin is based on the interpretation of ultrasound and MRI images, and postnatally on the histopathological evaluation of the surgical specimens [18]. In newborns, the incidence of pancreatic and hepatic tumor formations is extremely rare, making them difficult to diagnose and manage adequately (Table 5).

The rate of antenatal diagnosis of fetal intra-abdominal tumors is unknown due to the low number of cases. The use of ultrasound screening during pregnancy allowed the detection of intra-abdominal tumor masses [20]. The ultrasound appearance is similar for both malignant and benign fetal tumors, rarely showing a distortion of the surrounding anatomical structures or a specific Doppler pattern [21]. Thus, the suspicion of malignancy is extremely difficult to achieve.

Prenatal ultrasound diagnosis of congenital abdominal tumors is difficult to achieve depending on the tumor’s gestational age, origin, and characteristics. The use of 3D/4D reconstruction techniques, TUI Doppler, helps diagnose these tumor masses and fetal MRI, further contributing to establishing the relationship of the tumor with neighboring organs. The evaluation of the association of these congenital anomalies includes fetal MRI to increase the accuracy of the diagnosis, make a differential diagnosis, highlight the location, and establish the tumor morphology and the relationship with the adjacent structures, allowing the correct preoperative assessment and sometimes the prognosis of these tumors [19]. Computed tomography may differentiate between hepatic, adrenal, pancreatic hamartoma, and malignant lesions in some difficult cases. The final diagnosis will be confirmed histologically [18].

Prenatal management of the fetus requires a detailed ultrasound evaluation to detect any associated malformations and sometimes genetic evaluation [18]. Of the three cases of pancreatic hamartoma reported in newborns, one was diagnosed prenatally [15], one postnatally [13], and one at autopsy [7]. In 2000, Sepulveda et al. [15] reported the first prenatal sonographic diagnosis of a pancreatic tumor at 27 weeks of gestation; in our study, we identified at 25 weeks a pancreatic echogenic mass, relatively homogeneous, and IVC compression by the tumor.

Neonatal hamartomas are generally rare, with a higher kidney incidence. However, cases involving the liver, lungs and heart, brain, skeletal system, and gastrointestinal tract have been described in the literature. For example, a study by Kamil et al. showed on a batch of 84 tumors that only 19% had an intra-abdominal origin, with 15.4% solid tumors [22]. On the other hand, Amari et al., in a retrospective study of 354 fetuses, found that 6.2% were solid tumors, of which only one was a hamartoma and none a hepatoblastoma [23].

Prenatally, monitoring of fetal condition, tumor growth rate, and compression of adjacent organs are elements that usually do not significantly influence the decision and the method of birth in the fetus’s interest. A rapid increase in abdominal circumference secondary to intra-abdominal tumor volume, Doppler changes, or prenatal detection of tumor formation increase the rate of elective cesarean section [24]. Postnatally, the optimal operative moment regarding the complete resection of the tumor depends on the newborn’s general condition to avoid its rapid deterioration [25].

The synchronous presence of pancreatic and liver tumors in the newborn is unique and without a similar correspondence in the literature. The histopathological results raised many initial elements of confusion, which also influenced therapeutic management. According to the case definitions, the histopathological examination of specimens and the immunohistochemistry analysis diagnosed pancreatic hamartoma and hepatoblastoma.

In the case of hamartomas, the changes can be observed either mesenchymally or epithelially secondary to stromal induction, part of the proliferative lesion. As a result, cytogenetic or molecular abnormalities may explain the possible appearance of malignant neoplasms [26].

A study has shown a risk factor for hepatoblastoma associated with very low preterm birth (VLBW) [27]. Hepatoblastoma has a genetic component and is associated with Beckwith–Weidemann syndrome and familial adenomatous polyposis [28,29,30]. Ziogas et al. showed an increased incidence in children under 3 years of age (77.8%), the presence of vascular invasion in 27% of cases, and low survival in the absence of chemotherapy. Surgery combined with cisplatin-based chemotherapy resulted in the long-term survival of approximately 80% in hepatoblastoma [31]. The delay in diagnosing hepatoblastoma was due to the fact that the newborn was 36 weeks old (not a VLBW), AFP was normal, and the initial liver tumor was interpreted as a metastasis of the pancreatic tumor.

Most patients are asymptomatic as in our case; others may have abdominal discomfort, digestive disorders, and weight loss. The differential diagnosis of fetal intra-abdominal tumors is made in the order of their frequency with teratomas (large tumors, heterogeneous in appearance, showing cystic areas and sometimes calcifications), with polycystic kidney disease and adrenal (neuroblastoma), liver, pancreatic, gastrointestinal tract, and splenic tumors [1,23,32].

Rare tumors are usually misdiagnosed or late-diagnosed, the diagnosis being indirectly supported by the effect of the tumor on adjacent organs [23]. In our case, dilation of the IVC raised the suspicion of an abdominal tumor. Furthermore, modified insulin secretion of islet cell tissue secondary to pancreatic lesions is usually associated with modified levels of glycemia, but not in our patient.

In Table 4, tumor size varied from 1.2 to 14 cm by analyzing the cases presented, including ours. Three out of the ten tumors were located in the head of the pancreas, two were located in the tail, and three were diffusely present throughout the pancreas.

Imaging diagnosis of pancreatic tumors is important because the treatment options and prognosis are different. The surgical procedure is aimed at symptomatic pancreatic hamartomas and neuroendocrine tumors. Fetal abdominal tumors benefit from fetal intervention and access to neonatal surgery after birth. The presence of circulatory changes in the IVC is secondary to the progressive compression caused by the tumor with the progression of the tumor mass.

Three patients underwent a pancreaticoduodenectomy (PD)—Whipple operation, three a local resection, and one excision of the tumor with partial duodenectomy. Patients with pancreatic hamartoma did not undergo any type of adjuvant chemotherapy. In our case, the patient was initially diagnosed with serous cystadenocarcinoma and received at 2 months old a round of chemotherapy. No data have been reported on the recurrence or metastasis of pancreatic hamartomas.

The association of pancreatic hamartoma with hepatoblastoma and extensive thrombosis of the IVC and renal veins has led to poor survival.

Antenatal evaluation is important to determine the time of birth and subsequent therapeutic conduct. Therefore, managing these tumors involves a multidisciplinary team of obstetricians, neonatologists, oncologists, surgeons, and pathologists.

## 4. Conclusions

This study shows the importance of early imaging diagnosis of tumor masses in the pancreas and liver in the fetal and neonatal period. The histological and immunohistochemical evaluation is the one that confirms the final diagnosis, with therapeutic management individualized through adequate prenatal counseling and multidisciplinary involvement. The synchronous presence of pancreatic hamartoma and hepatoblastoma was a feature of this case.

## Figures and Tables

**Figure 2:**
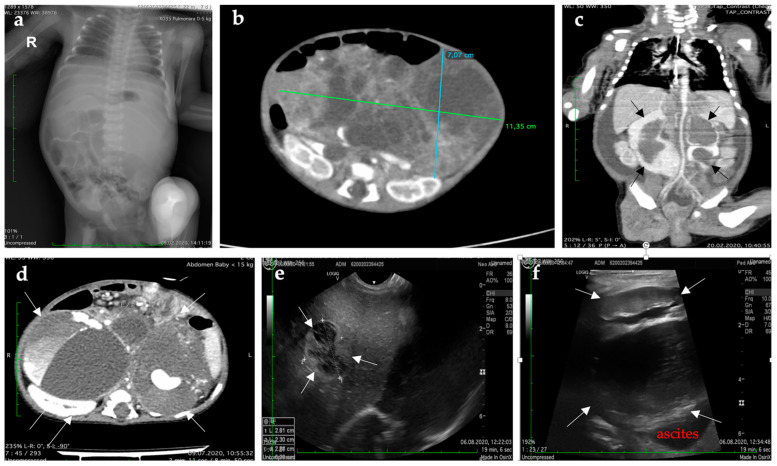
Postnatal imagistic findings. (**a**) Initial preoperative radiological evaluation showing ascension of the diaphragm due to pancreatic tumor mass. (**b**) Preoperative CT scan reveals a large mass in the fetal pancreas. (**c**) Postoperative CT scan showed residual tumor. (**d**) CT scan reveals multiple hepatic tumors with cystic components (arrows). (**e**) Ultrasonography showed liver relatively homogeneous with a nodular image of 23 mm with mixed appearance, predominantly transonic in segment VII. (**f**) Ultrasonography revealed multiple hepatic tumors with cystic components (arrows).

**Figure 4:**
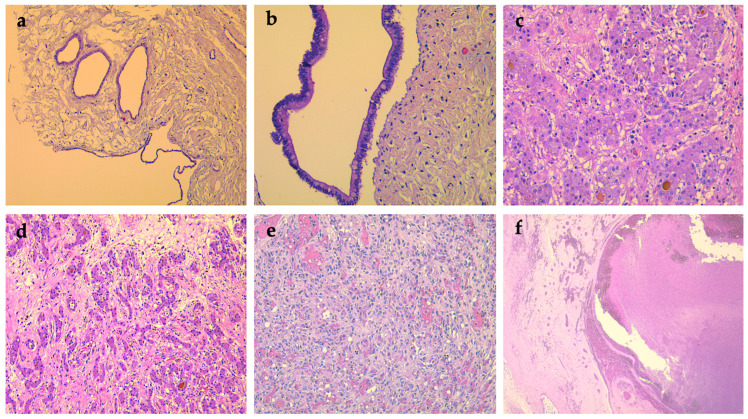
Histological findings of the pancreatic hamartoma, hepatoblastoma, and vena cava blocks. (**a**) Hamartoma of the pancreas: Cysts of variable sizes, lined by a single layer of cuboidal or columnar cells (H&E staining, ×100). (**b**) Hamartoma of the pancreas: Cyst with foveolar lining, punctuated by focal goblet-cell differentiation (H&E staining, ×200). (**c**) Hepatoblastoma: Nests of hepatocytes of the developing fetal liver, separated by fibrous stroma (H&E staining, ×100). (**d**) Hepatoblastoma: Thin trabeculae of hepatocytes forming pseudo acinar spaces with central bile plug (H&E staining, ×200). (**e**) Vessel wall: Granulation tissue with neoangiogenesis inside the vena cava wall (H&E staining, ×200). (**f**) Recent giant thrombus formed by fibrin and erythrocytes (H&E staining, ×25).

**Figure 5:**
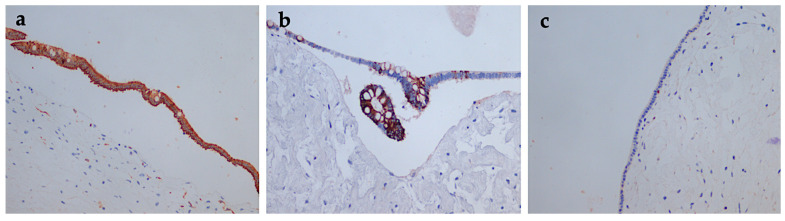
Immunohistochemical findings of the pancreatic hamartoma. (**a**) CK8/18 diffuse positive in the cysts epithelium, 200×; (**b**) CK20 focal positive in epithelial cells, 200×; (**c**) Low proliferative index—Ki67 (<1%) in the hamartomata’s epithelium, 200×.

**Table 1:** Synopsis of prenatal imagistic findings.

Type of Exam	Age	Imagistic Findings
Abdominal ultrasound	25th week	pancreatic echogenic mass (34 × 30 × 55 mm), relatively homogeneous; no Doppler blood flow lungs, liver, and kidneys—normal aspect amniotic fluid—normal arterial and ductus venosus Doppler—normal
Abdominal ultrasound	29th week	pancreatic echogenic mass (50 × 35 × 65 mm) liver and both kidneys—normal aspect compression of the inferior vena cava, with retrograde dilation thrombosis of the subhepatic segment of the IVC amniotic fluid—normal arterial and ductus venosus Doppler—normal

**Table 4:** Characteristics of pancreatic hamartomas of newborns and children reported in the all-time literature.

	Author (Year) [Ref]	Age	Sex	Clinical Features of the Tumor	Management and Prognosis
1	Smith (1960) [12]	2 m	F	Solid, tail	Trisomy 18
2	Rohde (1964) [11]	2 m	F	Cysts	Trisomy 18
3	Burt (1983) [13]	34 w	F	Solid and cystic, 11.5 cm, diffuse	PD and splenectomy/alive 3 m
4	Flaherthy (1992) [14]	20 m	F	Solid and cystic, 9 cm, head	Local resection/alive 9 m
5	Sepulveda (2000) [15]	27 w	M	Large multicystic, 12 cm, diffuse	Excision of the tumor and partial duodenectomy/at 1 year of age, he remains symptom-free
6	Thrall (2007) [16]	3 y	M	Multicystic adenomatoid, 3 cm, head	PD
7	Sueyoshi (2009) [17]	14 m	M	Multicystic adenomatoid, 14 cm, tail	Local resection/alive 26 m
8	Delgado (2017) [7]	33 w	F	Cysts, 1.2 cm	Trisomy 18/alive 1 h
9	Hosfield (2019) [8]	4 y	M	Multicystic adenomatoid, 9.5 cm, head	PD/alive 3 m
10	Present case (2020)	36 w	F	Multicystic adenomatoid, 11 cm, diffuse	Local resection/alive 11 m

PD—pancreaticoduodenectomy, F—female, M—male, m—months, w—weeks, y—years.

**Table 5:** Synopsis of imagistic evaluation of fetal pancreatic and liver tumors. Adapted from Birkemeier, K.L. (2020) [19].

	Imagistic Features	Prenatal Diagnosis
Pancreas		
Hamartoma	- hypoechoic mass with a clear boundary - on MRI, the shape of the tumor was often regular	2nd and 3rd trimesters
Adenocarcinoma	- hypovascular mass with pancreatic duct dilatation - invasion of surrounding tissues	NA
Neuroendocrine tumor	- solid, circumscribed mass, with hypervascular pattern - initial intensification after administration of the contrast substance - large tumors may show cystic areas, calcifications	NA
LIVER		
Hemangioma	- large, well-circumscribed tumor with variable echogenicity - T2-hyperintense mass on MRI, without pseudocapsule, with areas of necrosis and calcifications - at the periphery of the lesion, large vessels and areas of bleeding	3rd trimester
Mesenchymal hamartoma	- cystic and solid multilocular lesions with septa, nodules, and intracystic hemorrhage - peduncled or calcified - polyhydramnios	2nd and 3rd trimesters
Hepatoblastoma	- hyperechoic solid mass with hypervascularity - large solitary/multifocal heterogeneous mass - T2-hyperintense mass on MRI. - areas of hemorrhage or necrosis with compression of venous structures	late 3rd trimester

## Data Availability

The clinical data of the patient are available in the hospital database.
